# Adherence to the pro-inflammatory diet in relation to prevalence of irritable bowel syndrome

**DOI:** 10.1186/s12937-019-0487-6

**Published:** 2019-11-11

**Authors:** Asma Salari-Moghaddam, Ammar Hassanzadeh Keshteli, Ahmad Esmaillzadeh, Peyman Adibi

**Affiliations:** 1 Students’ Scientific Research Center, Tehran University of Medical Sciences, Tehran, Iran; 2 Department of Community Nutrition, School of Nutritional Sciences and Dietetics, Tehran University of Medical Sciences, P.O. Box 14155-6117, Tehran, Iran; 3 Department of Medicine, University of Alberta, Edmonton, Alberta Canada; 4 Integrative Functional Gastroenterology Research Center, Isfahan University of Medical Sciences, Isfahan, Iran; 5 Obesity and Eating Habits Research Center, Endocrinology and Metabolism Molecular-Cellular Sciences Institute, Tehran University of Medical Sciences, Tehran, Iran; 6 Department of Community Nutrition, School of Nutrition and Food Science, Isfahan University of Medical Sciences, Isfahan, Iran

**Keywords:** Dietary inflammatory index, DII, Inflammation, Irritable bowel syndrome, IBS

## Abstract

**Objective:**

There is no prior study that examined the association between nutrient-based dietary inflammatory index (DII) and odds of Irritable Bowel Syndrome (IBS). We examined the association between DII score and odds of IBS and its severity among Iranian adults.

**Methods:**

In this cross-sectional study, dietary intakes of 3363 Iranian adults were examined using a validated Dish-based 106-item Semi-quantitative Food Frequency Questionnaire (DS-FFQ). DII was calculated based on dietary intakes derived from DS-FFQ. IBS was assessed using a modified Persian version of Rome III questionnaire.

**Results:**

After adjustment for potential confounders, we found that participants in the highest quintile of DII score had greater chance for IBS compared with those in the lowest quintile (OR: 1.36; 95% CI: 1.03–1.80). By gender, we found a significant association between DII score and IBS among women (OR: 1.41; 95% CI: 1.00–2.00). By BMI status, overweight or obese (BMI ≥ 25 kg/m^2^) individuals in top quintile of DII score had greater odds for IBS than those in the bottom quintile (OR: 1.64; 95% CI: 1.07–2.53). No significant association was observed between a pro-inflammatory diet and severity of IBS symptoms.

**Conclusions:**

Consumption of a pro-inflammatory diet was associated with increased odds of IBS, in particular among women and those with BMI ≥ 25 kg/m^2^.

## Introduction

Irritable Bowel Syndrome (IBS) is one of the most common functional gastrointestinal disorders [1] which presents by abdominal pain and changes in bowel habits [2]. This condition affects 11% of worldwide population [3]. The high prevalence of IBS is also reported from Iran; such that, it has been shown that 1.1 to 25% of Iranian adults are affected by this condition [4].

The etiology of IBS is not well recognized; however, several factors including genetic susceptibility, female gender, family history and dietary factors might contribute to this condition [5–8]. Inflammation has been postulated to play a key role in pathology of IBS. Low grade inflammation contributes to the GI motor dysfunction and abdominal symptoms in patients with GI disorders. Low grade inflammation in the mucosal compartment of the gut can alter function in the underlying neuromuscular tissues from animal studies [9]. Individuals with IBS have been shown to have high levels of low-grade systemic inflammation [10]. Therefore, potential factors that increase systemic inflammation might be involved in the incidence and exacerbation of IBS symptoms. Among others, dietary factors are the most important one due to their unavoidable universal exposure to all people [11]. Dietary factors stimulating inflammatory process might be involved in the IBS pathology. To assess the inflammatory potential of the diet, recently Dietary Inflammatory Index (DII) has been constructed, which categorizes individual’s diet on a continuum from maximally anti-inflammatory to maximally pro-inflammatory [12]. The association between DII and inflammatory markers has been reported in earlier studies [13, 14]. Chronic pro-inflammatory conditions including obesity, metabolic syndrome and cardiovascular diseases have been extensively linked with DII [15–17]. However, no earlier study has assessed the association between DII and IBS. In a case-control study on patients with inflammatory bowel disease, consumption of pro-inflammatory diet has been associated with greater risk of inflammatory bowel disease [18]. In addition, adherence to western dietary pattern, which is mostly a pro-inflammatory diet, was prospectively associated with increased risk of IBS [19]. Consumption of fast food dietary pattern was also linked with greater odds of IBS [20].

The inflammatory potential of the diet can be examined by considering the anti- and pro-inflammatory properties of nutrients and foods. In a previous study, we developed an empirically-derived food-based dietary inflammatory index (FDII) and examined its association with IBS [21]. In that study, we found that consumption of a pro-inflammatory diet was associated with increased risk for IBS. However, nutrient-based DII might be differently associated with IBS and its severity because of the interactions among nutrients and their synergistic effects on each other in the gastrointestinal lumen. To our knowledge, there is no study on the association between nutrient-based DII and odds of IBS. In addition, most studies on the association between diet and IBS have focused on dietary components that alleviate IBS symptoms. Moreover, previous studies on the association of DII and chronic diseases have mostly focused on metabolic abnormalities rather than non-metabolic diseases. Finding the association of DII with non-metabolic conditions, including IBS, may help expanding the application of this index in dietary recommendations. This study was done to examine the association between DII score and IBS in a large sample of Iranian adults.

## Materials and methods

### Participants

This cross-sectional study was conducted within the framework of the Study on the Epidemiology of Psychological, Alimentary Health and Nutrition (SEPAHAN) project, a cross-sectional study that investigated the prevalence of functional gastrointestinal disorders (FGIDs) and their relationship with lifestyle factors. Details about SEPAHAN project have been published elsewhere [22]. Inclusion criteria for this study were as follow: Iranian general adults (aged 18–55 years) working in 50 different healthcare centers affiliated to Isfahan University of Medical Sciences (IUMS) across Isfahan province. In this project, data were collected in 2 main phases between April 2010 and May 2010. To collect information about anthropometric indices, demographic and lifestyle factors, including dietary intakes and physical activity, self-administered questionnaires distributed among 10,087 subjects in the first phase, and 8691 participants returned the completed questionnaires (response rate: 86.16%). In the second phase, data regarding gastrointestinal health were collected (response rate: 64.6%). Finally, we were able to match 4763 questionnaires in the second phase with their corresponding questionnaires in the first phase. In the current study, we excluded subjects who had total daily energy intakes outside the range of 800–4200 kcal/d as well as those that had missing data on any relevant variable. Therefore, data from 3363 subjects, for whom complete information about both dietary intakes and IBS were available, were included in the current analysis. All participants provided written inform consent forms. The study protocol was ethically approved by the Regional Bioethics Committee of Isfahan University of Medical Sciences.

### Dietary intakes assessment

Dietary data were collected using a Willett-format dish-based 106-item semi-quantitative food frequency questionnaire (DS-FFQ) which was designed and validated specifically for Iranian adults. Detailed information about the design, foods included, and the validity of this questionnaire has been published elsewhere [23]. Briefly, the questionnaire contained five categories of foods and dishes: 1) mixed dishes (cooked or canned, 29 items); 2) grains (different types of bread, cakes, biscuits and potato, 10 items); 3) dairy products (dairies, butter, and cream, 9 items); 4) fruits and vegetables (22 items); and 5) miscellaneous food items and beverages (including sweets, fast foods, nuts, desserts and beverages, 36 items). For each food item, a commonly consumed portion size was defined. Participants were asked to report their dietary intakes of foods and mixed dishes based on nine multiple choice frequency response categories varying from “never or less than once a month” to “12 or more times per day”. The frequency response categories for the food list varied from six to nine choices. For foods consumed infrequently, we omitted the high-frequency categories, while for common foods with a high consumption, the number of multiple choice categories increased. For instance, the frequency response for tuna consumption included six categories, as follows: never or less than once/month, 1–3 times/month, 1 time per week, 2–4 times/week, 5–6 times/week, 1–2 times/day; and for tea consumption that is highly prevalent among Iranians, the frequency response included nine categories, as follows: never or less than 1 cup/month, 1–3 cups/month, 1–3 cups/week, 4–6 cups/week, 1 cup/day, 2–4 cups/day, 5–7 cups/day, 8–11 cups/day, ≥12 cups/day). Finally, to convert the food items into grams, we computed the amount of each portion size based on the booklet of “household measures” and then computed the amount of intake by considering the frequency of consumption of each food item. The validity of DS-FFQ was examined in a subgroup of 200 randomly selected participants of SEPAHAN project. All participants in the validation study completed the DS-FFQ at study baseline and 6 months later. During this validation study, participants provided three detailed dietary records that were used as gold standard. As shown in earlier studies [23], it seems that this questionnaire provides reasonably valid measures of long-term dietary intakes.

### Assessment of dietary inflammatory index

Dietary data derived from DS-FFQ were used to calculate DII scores for all subjects. Earlier studies reported the development [12] and construct validation of the DII [24, 25]. Shivappa et al. [12] found that a total of 45 specific foods and nutrients were associated with one or more of the inflammatory [Interleukin-1β (IL-1β), Interleukin-6 (IL-6), Tumor Necrosis Factor-α (TNF-α) or CRP] or anti-inflammatory biomarkers [Interleukin-4 (IL-4) and Interleukin-10 (IL-10)]. Then, they scored the inflammatory potential for each food parameter according to whether it increased inflammatory or decreased anti-inflammatory factors (+ 1), or it decreased inflammatory or increased anti-inflammatory factors (− 1), or had no effect (0) on inflammatory or anti-inflammatory biomarkers. They calculated world mean and standard deviation for each of the 45 food parameters based on 11 data sets from 11 countries in different parts of the world. Due to lack of consumption of some foods in Iranian dietary culture as well as missing some items (like polyphenols) in our nutrient database, in the current study we calculated DII score based on 29 food parameters (rather than 45). The food parameters we used in the current study were as follow: pro-inflammatory parameters included energy, carbohydrate, fat, protein, cholesterol, saturated fat, trans fat, vitamin B12 and iron and anti-inflammatory parameters included mono-unsaturated fatty acids (MUFA), poly unsaturated fatty acids (PUFA), fiber, vitamin B6, folic acid, niacin, riboflavin, thiamin, vitamin A, vitamin C, vitamin D, vitamin E, β-carotene, caffeine, pepper, onion, tea, zinc, selenium, and magnesium. First, we calculated energy-adjusted amounts of these nutrients using residual method. Then, to calculate DII score for each participant, we calculated the *z* score for a given food parameter by subtracting the “standard global mean” from the amount consumed by each subject and dividing this value by the “global standard deviation”. Global means and standard deviations were obtained from the study of Shivappa et al. [12] We converted this value to a centered percentile score in order to reduce skewness, as earlier studies did [12]. For each participant, this score was then multiplied by the respective food parameter effect score derived from the study of Shivappa et al. [12]. Then, we calculated overall DII score for each participant by summing up all foods’ DII score. A higher DII score (more positive) indicates a more inflammatory diet and a lower DII score (more negative) indicates a less inflammatory diet. 

### Assessment of IBS

A modified Persian version of the Rome III questionnaire, as part of the main comprehensive questionnaire, was used for assessment of IBS. During the face validation of the questionnaire, we found that most participants were unable to distinguish between the descriptors used in the original Rome III questionnaire (never, less than 1 day a month, 1 day a month, 2–3 days a month, 1 day a week, more than 1 day a week, every day). We, therefore, modified the rating scales to consist of only four descriptors (i.e., never or rarely, sometimes, often, and always). We also decided to ask about the presence of each symptom in the past 3 months instead of questioning patients about the beginning of each symptom in more than 6 months prior to the evaluation, which already exists in original ROME III questionnaire. IBS was defined according to ROME III criteria as having recurrent abdominal pain or discomfort, at least sometimes, in the last 3 months associated with two or more of these criteria: improvement with defecation at least sometimes and onset associated with change in frequency or form (appearance) of stool, at least sometimes. IBS with constipation was defined as having IBS and both of the following criteria: (i) hard or lumpy stools at least sometimes and (ii) lack of loose, mushy or watery stools. IBS with diarrhea was defined as having IBS and both of the following criteria: (i) lack of hard or lumpy stools and (ii) loose, mushy or watery stools at least sometimes. Mixed IBS was defined as having IBS and both of the following criteria: (i) hard or lumpy stools at least sometimes and (ii) loose, mushy or watery stools at least sometimes. The severity of IBS was examined by asking participants on the severity of their abdominal pain in the last 3 months. They were able to choose one of these responses: mild/moderate/severe and very severe.

### Assessment of other variables

Required information on other variables including age, sex, marital status, smoking status, medication use and disease history (diabetes and colitis) was obtained from demographic and medical history questionnaires. Physical activity was assessed using the General Practice Physical Activity Questionnaire (GPPAQ) [26], and participants were classified into two categories: physically active (≥1 h/week) and physically inactive (< 1 h/week). Although this level of activity might seem low, but earlier publications have revealed that even 1 h per week of walking can reduce the risk of chronic conditions [27]. Data on diet-related practices including meal regularity (often or always/never or occasionally), chewing efficiency (a lot/not a lot) and intra-meal fluid intake (< 3 glasses/≥3 glasses) were also assessed through the use of a pretested questionnaire. Dental status was also examined and subjects were categorized as “having all teeth”, “lost 1-5 teeth” and “lost >5 teeth”. Anthropometric measures including weight, height, and waist circumference were assessed using a self-administered questionnaire. The validity of self-reported values of weight, height, and waist circumferences (WC) was examined in a pilot study on 200 participants from the same population. In the validation study, self-reported values of anthropometric indices were compared with actually measured values. The correlation coefficients for self-reported weight, height, and WC versus corresponding measured values were 0.95 (*P* < 0.001), 0.83 (*P* < 0.001), and 0.60 (*P* < 0.001), respectively. Body Mass Index (BMI) was calculated by dividing weight (kg) to height (m^2^). The correlation coefficient for computed BMI from self-reported values, and the one from measured values was 0.70 (*P* < 0.001).

### Statistical methods

We classified participants based on quintile cut-off points of DII score. General characteristics of study participants across quintiles of DII score were presented as means ±SDs for continuous variables and percentages for categorical variables. To examine the differences across quintiles, we used ANOVA for continuous variables and chi-square test for categorical variables. Energy-adjusted dietary intakes of study participants across quintiles of DII score were compared by using analysis of covariance (ANCOVA). We used binary logistic regression to estimate ORs and 95% CIs for the presence of IBS and its subtypes across quintiles of DII score in crude and multivariable-adjusted model. The trend of ORs across quintiles of DII score was determined by considering quintiles of DII score as ordinal variables in the logistic regression analysis. We also used multivariable ordinal logistic regression to estimate ORs and 95% CIs for assessing IBS severity (mild/moderate/severe/very severe) across quintiles of DII score in crude and multivariable-adjusted model. In these analyses, sex (male/female), smoking status (non-smoker/former smokers and current smokers), physical activity (< 1 h/week/≥1 h/week), medication use (yes/no), self-reported diabetes (yes/no) and colitis (yes/no), meal regularity (often or always/never or occasionally), chewing sufficiency (a lot/not a lot), intra-meal fluid consumption (< 3 glasses/≥3 glasses), and dental status (have all teeth/lost 1–5 teeth/lost > 5 teeth) were adjusted for in the multivariable model. All statistical analyses were done using the Statistical Package for Social Sciences (version 20; SPSS Inc.). *P* < 0.05 was considered as statistically significant.

## Results

In the current study, the DII score ranged from − 4.49 to + 5.39. Median overall DII score across increasing quintiles were − 2.05, − 0.90, − 0.02, + 0.86 and + 2.06, respectively. Overall, 22.2% of study participants had IBS (*n* = 748). General characteristics of study subjects are presented in Table 1. Compared with those in the lowest quintile, participants in the highest quintile of DII score were younger, less likely to be females, overweight, physically active and to have disease history of diabetes and colitis and more likely to have psychological distress. No significant differences were observed in terms of other variables across quintiles of DII score.

**Table 1:** General characteristics of study participants across quintiles of DII score^a^

Quintiles of DII score				
Variables	Q_1_	Q_3_	Q_5_	*P*-value^b^
DII range	−4.49 to −1.41	− 0.47 to + 0.44	+ 1.38 to + 5.39	
Subjects, n	672	672	672	
Age, y	37.7 ± 7.9	36.8 ± 7.7	34.9 ± 7.6	< 0.001
BMI, kg/m^2^	25.4 ± 3.9	25.04 ± 3.7	24.2 ± 3.6	< 0.001
Psychological distress	1.74 ± 2.38	1.93 ± 2.65	2.43 ± 2.91	< 0.001
Female, %	61.3	57.7	52.1	0.005
Married, %	81.7	83.4	79.9	0.62
Physically active, %	39.9	31.8	30.1	< 0.001
Current smokers, %	3.7	3.2	3.8	0.51
Disease history, %	5.8	4.3	4.8	0.04
Regular meal pattern, %				0.18
Often or always	62.8	62	57.3	
Never or occasionally	37.2	38	42.7	
Chewing sufficiency, %				0.36
A lot	14.3	14.2	11	
Not a lot	85.7	85.8	89	
Fluid consumption, %				0.72
< 3 glasses	97.2	96.1	96.5	
≥ 3 glasses	2.8	3.9	3.5	
Tooth loss, %				0.11
Have all	32.6	30.6	37.4	
Lost 1–5 teeth	58.1	62.5	55.1	
Lost >5 teeth	9.3	6.9	7.5	

^a^Data are mean ± standard deviation (SD)

^b^Obtained from ANOVA or chi-square test, where appropriate

A greater DII score was significantly associated with higher intakes of energy, carbohydrates, saturated fats, trans fats, niacin, thiamin and caffeine and lower intakes of fats, proteins, dietary fiber, cholesterol, MUFA, PUFA, vitamin B12, vitamin B6, folic acid, riboflavin, vitamin A, vitamin C, vitamin D, vitamin E, β-carotene, pepper, onion, tea, zinc and magnesium (average consumption of these parameters has been published earlier) [28].

**Fig. 1:**
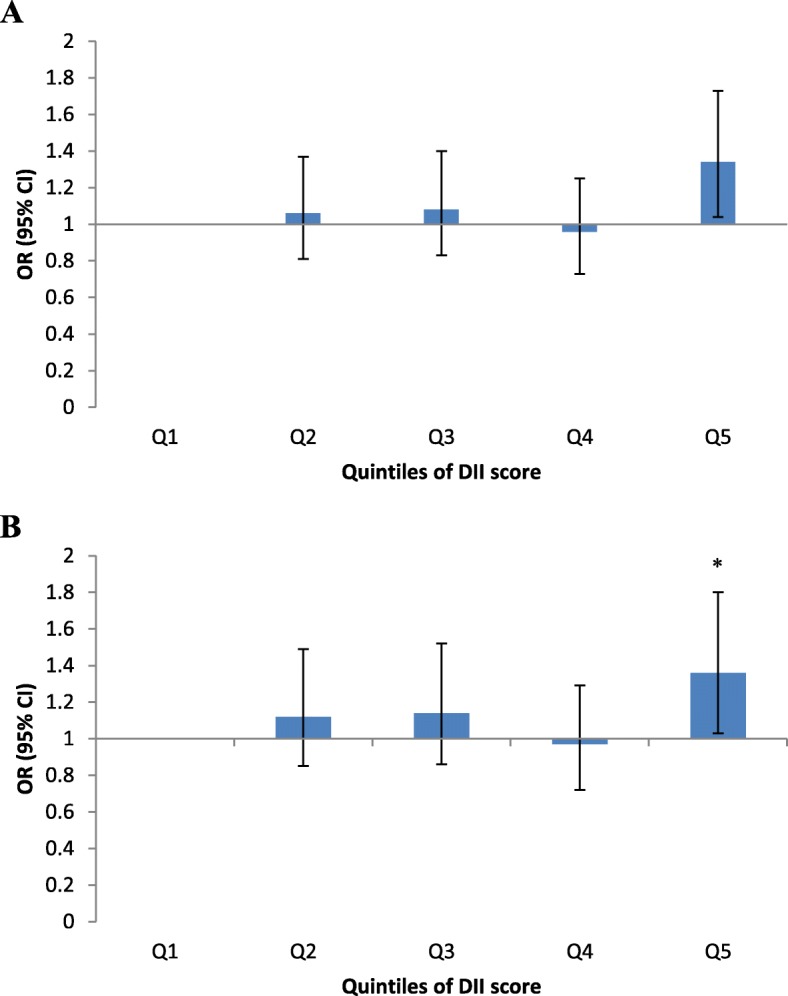
**a** The association between DII score and IBS in whole population (crude model). **b** The association between DII score and IBS in whole population (multivariable-adjusted model); ^*^P < 0.001 compared with the first quintile

In the crude model, participants in the highest quintile of DII score had greater chance for IBS (OR: 1.34; 95% CI: 1.04–1.73) compared with those in the lowest quintile (Fig. 1a). The association remained significant even after adjustment for potential confounders (OR: 1.36; 95% CI: 1.03–1.80) (Fig. 1b).

Gender- and BMI-stratified analysis on the association of DII score and prevalent IBS are provided in Table 2. When the analyses were done separately by gender, we found no significant association between DII score and IBS in men; however, among women, we observed a significant association between DII score and IBS in crude (OR: 1.54; 95% CI: 1.12–2.13) and multivariable-adjusted model; such that those in the highest quintile of DII score were 41% more likely to have IBS than those in the lowest quintile after controlling for potential confounders (OR: 1.41; 95% CI: 1.00–2.00). By BMI status, overweight and obese (BMI ≥ 25 kg/m^2^) individuals in top quintile of DII score had greater odds for IBS than those in bottom quintile (OR: 1.64; 95% CI: 1.07–2.53). However, we failed to find any significant association between DII score and IBS in normal weight (BMI < 25 kg/m^2^) individuals either before (OR: 1.37; 95% CI: 0.95–1.96) or after controlling for potential confounders (OR: 1.22; 95% CI: 0.83–1.80).

**Table 2:** Gender- and BMI-stratified odds ratios and 95% CIs for IBS across quintiles of DII score^a^

Quintiles of DII score				
	Q_1_	Q_3_	Q_5_	
Median DII	−2.05	−0.02	+ 2.06	*P*-trend
Male				
Crude	1.00	1.35 (0.87–2.08)	1.22 (0.80–1.88)	0.46
Multivariable-adjusted^b^	1.00	1.53 (0.94–2.48)	1.33 (0.82–2.16)	0.39
Female				
Crude	1.00	0.96 (0.69–1.34)	1.54 (1.12–2.13)	0.04
Multivariable-adjusted^b^	1.00	0.99 (0.70–1.41)	1.41 (1.00–2.00)	0.22
BMI < 25 (kg/m^2^)				
Crude	1.00	1.08 (0.73–1.59)	1.37 (0.95–1.96)	0.08
Multivariable-adjusted^c^	1.00	1.06 (0.70–1.59)	1.22 (0.83–1.80)	0.45
BMI ≥ 25 (kg/m^2^)				
Crude	1.00	1.15 (0.80–1.66)	1.41 (0.96–2.07)	0.27
Multivariable-adjusted^c^	1.00	1.32 (0.88–1.98)	1.64 (1.07–2.53)	0.09

^a^Values are OR (95% CIs). IBS was defined as having recurrent abdominal pain or discomfort at least sometimes in the last 3 months associated with two or more of these criteria: improvement with defecation at least sometimes and onset associated with change in frequency or form (appearance) of stool at least sometimes

^b^Adjusted for physical activity, smoking status, medication use, disease history (diabetes, colitis), psychological distress, regular meal pattern, chewing sufficiency, fluid consumption and dental status

^c^Adjusted for sex, physical activity, smoking status, medication use, disease history (diabetes, colitis), psychological distress, regular meal pattern, chewing sufficiency, fluid consumption and dental status

Crude and multivariable-adjusted ORs and 95% CIs for IBS severity across quintiles of DII score are presented in Table 3. No significant association was observed between DII score and IBS severity in crude (OR: 1.23; 95% CI: 0.77–1.96) or multivariable-adjusted model (OR: 1.08; 95% CI: 0.65–1.80) in subjects with IBS. This was also the case when we analyzed data separately by gender or BMI status.

**Table 3:** Odds ratios and 95% CIs for IBS severity across quintiles of DII score^a^

	Q_1_	Q_3_	Q_5_
Median DII	−2.05	−0.02	+ 2.06
Whole population			
Crude	1.00	0.97 (0.59–1.60)	1.23 (0.77–1.96)
Multivariable-adjusted^b^	1.00	0.90 (0.53–1.55)	1.08 (0.65–1.80)
Male			
Crude	1.00	0.67 (0.27–1.66)	1.34 (0.59–3.04)
Multivariable-adjusted^c^	1.00	0.57 (0.19–1.66)	1.11 (0.42–2.91)
Female			
Crude	1.00	1.18 (0.64–2.16)	1.18 (0.67–2.08)
Multivariable-adjusted^c^	1.00	1.11 (0.59–2.12)	1.07 (0.58–1.97)
BMI < 25 (kg/m^2^)			
Crude	1.00	0.70 (0.33–1.47)	0.81 (0.41–1.60)
Multivariable-adjusted^b^	1.00	0.73 (0.33–1.61)	0.79 (0.38–1.64)
BMI ≥ 25 (kg/m^2^)			
Crude	1.00	1.32 (0.64–2.70)	1.70 (0.85–3.38)
Multivariable-adjusted^b^	1.00	1.12 (0.50–2.51)	1.52 (0.70–3.32)

^a^Values are OR (95% CIs). IBS was defined as having recurrent abdominal pain or discomfort at least sometimes in the last 3 months associated with two or more of these criteria: improvement with defecation at least sometimes and onset associated with change in frequency or form (appearance) of stool at least sometimes

^b^Adjusted for sex, physical activity, smoking status, medication use, disease history (diabetes, colitis), psychological distress, regular meal pattern, chewing sufficiency, fluid consumption and dental status

^c^Adjusted for physical activity, smoking status, medication use, disease history (diabetes, colitis), psychological distress, regular meal pattern, chewing sufficiency, fluid consumption and dental status

Crude and multivariable-adjusted ORs and 95% CIs for IBS subtypes across quintiles of DII score are presented in Table 4. After controlling for potential confounders, we found that those with the greatest DII score had higher odds of IBS-M than those with the lowest DII score (OR: 1.90; 95% CI: 1.00–3.59). However; after adjustment for potential confounders, this association became non-significant (OR: 1.65; 95% CI: 0.84–3.23). No other overall association was seen between DII score and other types of IBS.

**Table 4:** Odds ratios and 95% CIs for IBS subtypes across quintiles of DII score^a^

	Quintiles of DII score			
	Q_1_	Q_3_	Q_5_	
Median DII	−2.05	−0.02	+ 2.06	*P*-trend
IBS-C				
Crude	1.00	0.84 (0.55–1.26)	1.12 (0.76–1.64)	0.24
Multivariable-adjusted^b^	1.00	0.88 (0.57–1.37)	1.15 (0.75–1.75)	0.29
IBS-D				
Crude	1.00	0.90 (0.53–1.50)	1.33 (0.83–2.13)	0.45
Multivariable-adjusted^b^	1.00	0.95 (0.54–1.66)	1.37 (0.81–2.30)	0.49
IBS-M				
Crude	1.00	2.33 (1.25–4.32)	1.90 (1.00–3.59)	0.16
Multivariable-adjusted^b^	1.00	2.31 (1.21–4.39)	1.65 (0.84–3.23)	0.25
IBS-U				
Crude	1.00	1.02 (0.65–1.61)	1.19 (0.76–1.85)	0.96
Multivariable-adjusted^b^	1.00	1.06 (0.65–1.72)	1.21 (0.75–1.94)	0.92

^a^Values are OR (95% CIs). IBS was defined as having recurrent abdominal pain or discomfort at least sometimes in the last 3 months associated with two or more of these criteria: improvement with defecation at least sometimes and onset associated with change in frequency or form (appearance) of stool at least sometimes

IBS-C: IBS with constipation; IBS-D: IBS with diarrhea; IBS-M: mixed IBS; IBS-U: unsubtyped IBS

^b^Adjusted for sex, physical activity, smoking status, medication use, disease history (diabetes, colitis), psychological distress, regular meal pattern, chewing sufficiency, fluid consumption and dental status

## Discussion

In this cross-sectional study, we found that adherence to a pro-inflammatory diet was associated with increased odds of IBS in the whole population as well as in women, but not in men. There was a significant association between DII score and IBS in overweight and obese (BMI ≥ 25 kg/m^2^) subjects; however, the association was not significant in normal weight (BMI < 25 kg/m^2^) participants. No significant association was observed between adherence to a pro-inflammatory diet and odds of IBS severity.

Irritable bowel syndrome is one of the most common gastrointestinal disorders [1]. Preventive strategies including lifestyle modifications are of great importance in this regard. Diet and inflammation have been proposed to play an important role in the etiology of IBS. We found a positive significant association between inflammatory potential of the diet and odds of IBS. To our knowledge, this is the first study that examined the association between a pro-inflammatory diet and IBS. It is well established that IBS is an inflammatory condition [9]. Therefore, it is expected that the pro-inflammatory potential of the whole diet might be associated with IBS. It has been reported that IBS patients have lower intakes of fruits, vegetables and dairy products [29], all with anti-inflammatory properties. There is no previous study that examined the association of DII score and IBS; however, the association of this index with other pro-inflammatory conditions has been examined. In a case-control study, the investigators reported a significant association between DII score and ulcerative colitis [18]. The association of DII with diabetes, metabolic syndrome and obesity has also been observed in earlier studies [15, 16]. These findings suggest that DII may elucidate the role of diet in the development of chronic diseases through inflammatory process.

We observed a gender difference in the association of a pro-inflammatory diet and IBS. Previous studies showed a higher prevalence of IBS among women than men [30]. These findings suggest a role for sex hormones in the etiology of IBS. Sex hormones may modulate IBS onset and exacerbation. In addition, slow GI transit, delayed gastric empty, and reduced colonic transit time among women than men might be mediated, at least in part, by sex hormones [31]. On the other hand, stress, a major contributing factor to IBS, is highly prevalent among women than in men [32]. Given the elevated levels of inflammation in stressed subjects and the probable interaction of diet with these situations, one might explain the gender difference in the association of pro-inflammatory potential of the diet and IBS.

We found a significant direct association between a pro-inflammatory diet and IBS among overweight and obese participants, but not in normal-weight subjects. This observation was in line with earlier studies that showed a higher prevalence of IBS in overweight and obese individuals compared with normal-weight subjects. A cross-sectional study that examined the association between gastrointestinal symptoms and BMI, reported a significant association between obesity and IBS symptoms [33]. Another study reached the same findings [34]. It is well known that overweight and obesity are associated with elevated levels of circulating inflammation [35]. High prevalence of IBS in obese subjects might also be explained by inflammation. Given the inflammatory nature of obesity and IBS, consumption of a pro-inflammatory diet might exacerbate these conditions.

We observed no significant association between a pro-inflammatory diet and IBS severity in the whole population as well as among overweight and obese subjects. Previous studies have shown a low-grade intestinal inflammation in IBS patients and consequent increased intestinal permeability [36]. A cross-sectional study showed higher prevalence of functional bowel symptoms in patients with morbid obesity [37]. Another cross-sectional study in Sweden reported greater severity of symptoms in overweight and obese subjects with IBS compared with normal weight subjects with IBS [38]. Such association has been shown in other studies as well [39, 40]. It has been shown that elevated inflammation would aggravate the IBS symptoms. Therefore, given the nature of irritable bowel syndrome, association between adherence to a pro-inflammatory diet and odds of IBS severity is expected. Lack of finding a significant association in the current study might be attributed to the assessment of IBS severity by only a question. It seems that accurate assessment of IBS severity needs further investigation and the severity of abdominal pain only, which was used in this study, might not reflect IBS severity. In addition, having low number of patients with severe IBS in the current study might provide another reason. Most patients in the study have indicated that they have mild to moderate abdominal pain. Further studies with prospective design are warranted to shed light on this issue.

There are several mechanisms through which a pro-inflammatory diet might influence IBS. Patients with IBS have higher levels of inflammatory cytokines. A pro-inflammatory diet can increase systemic inflammation [13]; therefore, it might be involved in the incidence and exacerbation of IBS symptoms. Consumption of pro-inflammatory diet has been linked with obesity [15]. Small bowel and colonic transit alteration in obese subjects might also explain IBS symptoms [41].

## Conclusion

Our study has several strengths. This is the first study that examined the association of a pro-inflammatory diet and odds of IBS. Large sample size and taking the role of potential confounders into account are among other strengths of the present study. In addition, dietary habits which contribute to gastrointestinal disorders were considered as covariates in our analyses. Some limitations should also be considered when interpreting our findings. First, due to cross-sectional nature of the present study, causal relationships between DII score and IBS cannot be established. Therefore, further studies especially with prospective design are warranted to confirm our findings. Second, although we controlled for several potential confounders, the possibility of residual confounding cannot be excluded. In the current study, we used a validated DS-FFQ for dietary assessment and DII score calculation; however, measurement errors and misclassification of study participants cannot be avoided. In addition, some parameters were missing for DII score calculation due to lack of data on some anti-inflammatory parameters including alcohol, eugenol, garlic, ginger, n-3 fatty acids, n-6 fatty acids, saffron, turmeric, flavan-3-ol, flavones, flavonols, flavonones, anthocyanidins, isoflavones, thyme/oregano and rosemary; therefore, we did not consider these dietary parameters in the calculation of DII score. The DII used in this study was based on earlier studies and it was not validated in Iranian population to examine whether it can really predict inflammation. However, based on the application of this score for prediction of different inflammatory-related conditions in the country, it seems that this index is a valid tool. For identification of IBS, we used questionnaire-based data. Although the validity of Rome III questionnaire has been shown in Iranian adults, the possibility of misclassification in terms of having IBS cannot be avoided.

In conclusion, we found that consumption of a pro-inflammatory diet was associated with increased odds of IBS. A significant association between DII score and IBS was observed in women, but not in men. In addition, a significant positive association was seen between DII score and IBS in overweight and obese subjects, but not in normal-weight participants. Further studies, especially with prospective design, are required to confirm our results.

## Data Availability

The dataset used and analyzed during the current study is available from the corresponding author on a reasonable request.

## Abbreviations

BMI: Body Mass Index
CI: Confidence Interval
DII: Dietary Inflammatory Index
DS-FFQ: Dish-based Semi-quantitative Food Frequency Questionnaire
FDII: Food-based Dietary Inflammatory Index
FGIDs: Functional Gastrointestinal Disorders
GPPAQ: General Practice Physical Activity Questionnaire
IBS: Irritable Bowel Syndrome
OR: Odds Ratio
SEPAHAN: Study on the Epidemiology of Psychological, Alimentary Health and Nutrition
WC: Waist Circumference
